# Therapeutic effects of recombinant human interleukin 2 as adjunctive immunotherapy against tuberculosis: A systematic review and meta-analysis

**DOI:** 10.1371/journal.pone.0201025

**Published:** 2018-07-19

**Authors:** Ruimei Zhang, Xiangyu Xi, Chunying Wang, Yong Pan, Changhua Ge, Liying Zhang, Shuo Zhang, Huimei Liu

**Affiliations:** Department of Tuberculosis, Xuzhou Infectious Disease Hospital, Xuzhou, Jiangsu Province, PR China; University of Washington, UNITED STATES

## Abstract

**Background:**

Interleukin 2 (IL-2) is a cytokine secreted by activated T cells. Studies exploring recombinant human interleukin 2 (rhuIL-2) as an adjunctive immunotherapeutic agent to treat tuberculosis (TB) have shown variable results; however, the true therapeutic efficacy of rhuIL-2 administration in TB patients has not been determined.

**Methods:**

A systematic review to identify publications exploring the association between rhuIL-2-based immunotherapy for TB and outcomes (sputum culture conversion, sputum smear conversion, radiographic changes, and leukocyte phenotype changes) in patients with pulmonary TB published before June 8, 2018 was performed. Data were extracted and analyzed by two investigators independently.

**Results:**

A total of 2,272 records were screened. Four randomized controlled trials (RCTs) comprising 656 pulmonary TB patients were finally included. The rhuIL-2 treatment could significantly improve the sputum culture conversion of TB (RR, 1.18; 95%CI: 1.03–1.36; I^2^ < 0.01; P = 0.019) after at least 3 months of anti-TB therapy and the sputum smear conversion of TB during anti-TB therapy. Treating multidrug-resistant tuberculosis (MDR-TB) with rhuIL-2 could improve the sputum culture conversion (RR, 1.28; 95%CI: 1.05–1.57; I^2^ < 0.01; P = 0.016) and smear conversion (RR, 1.28; 95%CI: 1.09–1.51; I^2^ < 0.01; P = 0.003) at the end of anti-TB treatment. Meanwhile, rhuIL-2-based adjunctive immunotherapy could expand the proliferation and conversion of CD4^+^ and natural killer (NK) cells. Three of the included studies suggested that radiographic changes could not be improved by the use of rhuIL-2 as adjunctive immunotherapy. Publication bias did not exist.

**Conclusions:**

Based on this first meta-analysis, rhuIL-2-based adjunctive immunotherapy appears to expand the proliferation and conversion of CD4^+^ and NK cells, as well as improve the sputum culture (at 3 months and later) and smear conversion of TB patients.

## Introduction

Tuberculosis (TB) is the most common serious infectious disease and a global health concern caused by *Mycobacterium tuberculosis (M*. *TB)* or *M*. *bovis*. According to reports published by the World Health Organization (WHO), millions of new TB cases occur each year, causing almost two million deaths annually [1–3]. The occurrence of human immunodeficiency virus (HIV)-associated TB and the growing incidence of multidrug-resistant *M*. *TB* (MDR-TB) isolates have generated this emergency. Therefore, it is necessary to develop better control strategies.

For many years, TB has been effectively controlled and cured by the combined chemotherapy of isoniazid, rifampicin, pyrazinamide, and ethambutol, which is recommended by the WHO[4–6]. However, adverse events (e.g., long-term administration, toxicity, and intolerance) are always accompanied with the chemotherapy. On the other hand, MDR-TB, defined as isolates resistant to both isoniazid and rifampicin with or without resistance to other anti-TB drugs, causes significant problems and constitutes an increasing public health concern globally [7–9].

Interleukin 2 (IL-2), a cytokine secreted by activated T cells, promotes the differentiation and proliferation of lymphoid cells as well as enhances the cell-mediated immune response to infections [10]. Therefore IL-2 has been used as an adjunctive immunotherapeutic agent to control some infectious diseases, such as leishmaniasis, leprosy, and HIV infection [11–13]. Beginning in 1988, several studies have demonstrated that IL-2 administration in murine mycobacteria models could limit the course of mycobacterial infection [14–16]. In 1995, Johnson *et al*. found that recombinant human IL-2 (rhuIL-2) administration in combination with conventional multidrug therapy (MDT) is safe and may potentiate the antimicrobial cellular immune response to TB [17]. Meanwhile, several clinical studies have suggested that rhuIL-2 administered as an adjunct to conventional MDT to TB patients could induce immune activation and enhance the antimicrobial response, with significant improvement in the rate of conversion of sputum smears [18–20]. It also has been reported that IL-2 administration may increase T cell regulatory activity and facilitate bacilli proliferation [21]. However, others have reported that daily treatment with rhuIL-2 during the first month of TB management did not enhance bacillary clearance or improve symptoms in patients with drug-susceptible TB [22]. The balance between the management cost and patient benefits should also be carefully pondered [23]. Many reviews have paid attention to the use of rhuIL-2 as an adjunctive immunotherapeutic agent to treat TB [23–26]. However, the true therapeutic efficacy of rhuIL-2 administration in TB patients has not been determined.

Here, we report the systematic review and meta-analysis for the therapeutic efficacy of rhuIL-2 administration in the management of TB.

## Material and methods

This meta-analysis was performed following the guidelines of the Preferred Reporting Items for Systematic Reviews and Meta-analyses (PRISMA).

### Search strategy and data sources

The electronic databases (PubMed, Cochrane Library, EMBASE, and Web of Science) were searched by two investigators (HML and XYX) independently without language restriction from their inception until June 8, 2018. The search terms included “interleukin 2” OR “IL-2” OR “rhuIL-2” OR “recombinant human IL-2” AND “tuberculosis” OR “mycobacterium tuberculosis infection” OR “tuberculous lesion” OR “tuberculoses” OR “Kochs Disease” AND “randomized controlled study” OR “controlled clinical study” OR “randomized” OR “placebo” OR “randomly.”

### Study selection

The inclusion criteria for this study were as follows: (1) patients who were diagnosed with HIV-seronegative TB or with culture-confirmed *M*. *TB* or *M*. *bovis*; (2) rhuIL-2 was applied as adjunctive immunotherapy; (3) the clinical trial was a randomized controlled trial (RCT); (4) outcomes of the clinical trial included sputum culture, sputum smear, radiographic changes, and leukocyte phenotype changes.

The exclusion criteria for this study were as follows: (1) review, conference summary, or case report; (2) basic research; (3) studies involving children or pregnant woman; (4) duplicate studies.

### Data extraction

Data were extracted from the included studies by two investigators (CHG and SZ) independently. The first author, publication year, study population, age, sex ratio, dosage, time of rhuIL-2 use, sputum culture, sputum smear, radiographic changes, and leukocyte phenotype changes were collected using a predesigned electronic form.

Any key absent information was requested from the authors by e-mail. Studies were excluded if we did not get any response from the authors.

### Quality assessment

Two investigators (LYZ and HML) independently reviewed all of the studies and assessed the methodological quality and risk of bias for each eligible study by the Cochrane Collaboration tool [27]. The sequence generation, allocation concealment, blinding of participants and personnel, blinding of outcome assessment, incomplete outcome data, selective outcome reporting, and other potential bias were included.

### Statistical analyses

The data were analyzed using STATA v12.0 statistical software by two investigators (HML and RMZ) independently. For dichotomous variables (sputum culture, sputum smear, and radiographic changes), the relative risk (RR) and 95% confidence interval (CI) for each study were calculated. For continuous variables (leukocyte phenotype changes), the standard mean difference was calculated.

Heterogeneity was estimated by the I^2^ and the χ^2^ tests. The random-effects model was used when a significant heterogeneity (p ≤ 0.10 for the χ^2^ test or I^2^ ≥ 50%) was observed; otherwise, the fixed-effects model was applied. Both the Mantel-Haenszel test and inverse-variance weighting were used.

Publication bias was evaluated by a funnel plot. The z test was applied to determine the significance of the pooled index. *P* ≤ 0.05 was considered statistically significant.

## Results

### Literature search

**Fig 1:**
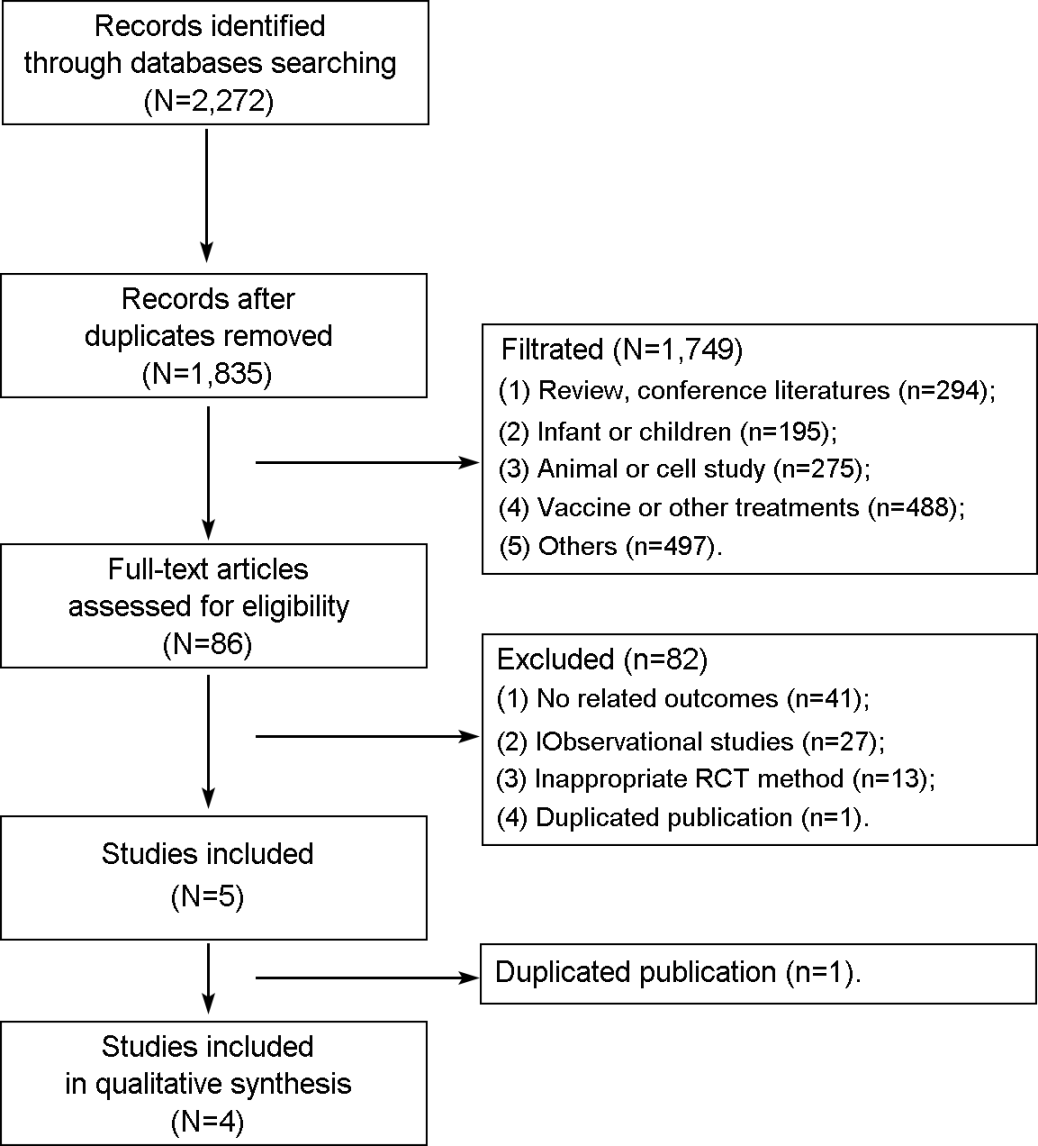
Flow diagram of the study selection process.

A total of 2,272 records were identified during the initial electronic database search. The information for primary exclusion is presented in Fig 1. After duplicates were removed, 1,835 records remained. Then, 1,749 records were excluded for different reasons, and 84 studies were analyzed. After reading the full texts, 81 studies were excluded, and 5 studies were included. However, the study performed by Shen *et al*. was also excluded because it was confirmed by one of the authors to be a duplicate study [20]. Finally, four studies (n = 656 patients) that met the inclusion criteria were included in this meta-analysis [28–30,18].

### Patient characteristics

The demographics of the patients are summarized in Table 1. All of the patients were HIV seronegative and had TB as determined by culture-confirmed *M*. *TB*. The study performed by Chu *et al*. did not provide the detailed demographic information of the included patients [30]. The mean age of the included patients in the other three studies ranged from 35.0 to 44.2 years. Patients were diagnosed with pulmonary TB [30,29] or MDR-TB, which was defined as culture-confirmed *M*. *TB* resistant to isoniazid and rifampicin [18,28]. However, 85 patients had MDR-TB in the study performed by Chu *et al*. [30]. Two studies focused on newly diagnosed TB [28,29], while the other two studies analyzed retreatment for TB [18,30]. There were 114 relapsing cases in the study performed by Tan *et al*. [28]. None of the included studies provided clear comorbidities. One study was conducted in Uganda [29], one study was conducted in South Africa [18], and two studies were from China [28,30].

**Table 1:** Patient demographics of the included trials.

Source	Tan *et al*. [28]	Johnson *et al*. [29]	Chu *et al*. [30]	Johnson *et al*. [18]
**Year of publication**	2017	2003	2003	1997
**Size (n)**	302	110	209	35
**Gender (M/F)**	168/134	75/35	Unclear	18/17
**Age (years)**	44.2	35.0 ^a^	Unclear	36.6
**Disease**	MDR-TB	TB	TB ^b^	MDR-TB
**Initial drug resistance**	Isoniazid and rifampin	No	No	Isoniazid and rifampin
**New or retreatment**	New, relapse	New	Retreatment	Retreatment
**HIV**	Seronegative	Seronegative	Seronegative	Seronegative
**Comorbidities**	Unclear	Unclear	Unclear	Unclear
**Center**	14 multicenter	Single center	Unclear	Single center
**Region**	China	Uganda	China	South Africa

M/F: male/female; TB: tuberculosis; MDR-TB: multidrug-resistant tuberculosis; HIV: human immunodeficiency virus.

^a^ median.

^b^ 85 patients were MDR-TB.

### Treatment protocols

All of the included patients were randomly divided into the intervention or control group. The protocols of treatment are shown in Table 2. All of the included patients received chemotherapy. The drug doses were adjusted according to the patient weight. Treatment assignments were masked to the clinical and laboratory staff.

**Table 2:** Protocols of rhuIL-2 treatment.

Source	Tan *et al*. [28]	Johnson *et al*. [29]	Chu *et al*. [30]	Johnson *et al*. [18]
**Year of publication**	2017	2003	2003	1997
**Intervention group**				
**-Source**	China	Canada	China	Canada
**-Beginning time**	After inclusion and allocation	After inclusion and allocation	After inclusion and allocation	After inclusion and allocation
**-Delivery method**	Subcutaneous injection	Intradermal injection	Intradermal injection	Intradermal injection
**-Bolus dose**	50 × 10^4^ U/mL	225,000 IU	200,000 IU	**DRG:** 225,000 IU **PRG:** 450,000 IU
**- Schedule**	Once every other day for 30 days separately during months 1, 3, 5, and 7.	Twice daily during the first 30 days of anti-TB treatment	Once daily for 30 days, followed by 30 days 'rest', for two cycles.	**DRG:** Twice daily for 30 consecutive days **PRG:** Daily at 12-h intervals for 5 days, followed by 9 days 'rest', for three cycles.
**-Therapy period**	7 months	30 days	90 days	30 days
**Control group**	Background drug regimen (chemotherapy)	Standard short-course chemotherapy with sterile 5% dextrose	Standard chemotherapy	Standard chemotherapy with diluent

TB: tuberculosis; DRG: Daily rhuIL-2 groups; PRG: Pulse-therapy rhuIL-2 group.

Patients in the control group received an optimized anti-TB chemotherapy regimen with or without diluents. Separate from the chemotherapy, patients in the rhuIL-2 group received rhuIL-2 treatment, after inclusion and allocation.

Patients in the intervention group were treated with rhuIL-2. However, the treatment protocols in the four studies were slightly different. First, the source of rhuIL-2 was different. Two studies used rhuIL-2 that was produced in China [30,28], while two studies used proleukin, which was from Chiron Corp, Canada [18,29]. Second, the method of rhuIL-2 delivery was different. One study adopted subcutaneous injection [28], and three studies used intradermal injection [20,29,18,30]. Third, the bolus dose was different. Two different bolus doses (225,000 IU and 450,000 IU) of rhuIL-2 were used in the study performed by Johnson *et al*. [18], while the other studies used a dose of 200,000 IU [30], 225,000 IU [29], or 50 × 10^4^ U/mL [28]. Fourth, the schedule of rhuIL-2 was different. Last, the length of rhuIL-2 therapy was different, ranging from 30 days to 8 months.

### Methodological quality and risk of bias

The quality and bias of the included studies are shown in Table 3. All studies were randomized. Both a computer-generated randomization sequence and a table of random numbers were adopted to avoid selection bias. Two studies did not report the method of sequence generation [20,28]. Two studies used a centrally assigned method and a block size of 10 to avoid allocation bias [29,28]. Two studies applied blinding methods [20,29]. Placebos were used as a control in all studies.

**Table 3:** Quality and bias of the included trials.

Source	Tan *et al*. [28]	Johnson *et al*. [29]	Chu *et al*. [30]	Johnson *et al*. [18]
**Year of publication**	2017	2003	2003	1997
**Selection bias**				
**-** Sequence generation	Unclear	Computer- generated	Computer-generated	Table of random numbers
**-** Allocation concealment	Centrally assigned method	Block size of 10	Low Risk	Low Risk
**Performance and detection bias**				
**-** Blinding of participants and personnel	Low Risk	Double-blind	High Risk	High Risk
**-** Blinding of outcome assessment	Low Risk	Double-blind	High Risk	High Risk
**Incomplete outcome data addressed**	Low Risk	Low Risk	Low Risk	Low Risk
**Selective reporting**	Low Risk	Low Risk	Low Risk	Low Risk
**Other bias**	Low Risk	Low Risk	Low Risk	Low Risk

### Sputum culture conversion

All four of the included studies reported the sputum culture, which was assessed at different stages (from 1 to 24 months) of anti-TB treatment [30,29,18,28]. Sputum samples were cultured by different methods, such as the BACTEC^TM^ broth culture system [18,29] and Lowenstein–Jensen solid medium in a Mycobacterium Growth Indicator Tube [28].

**Fig 2:**
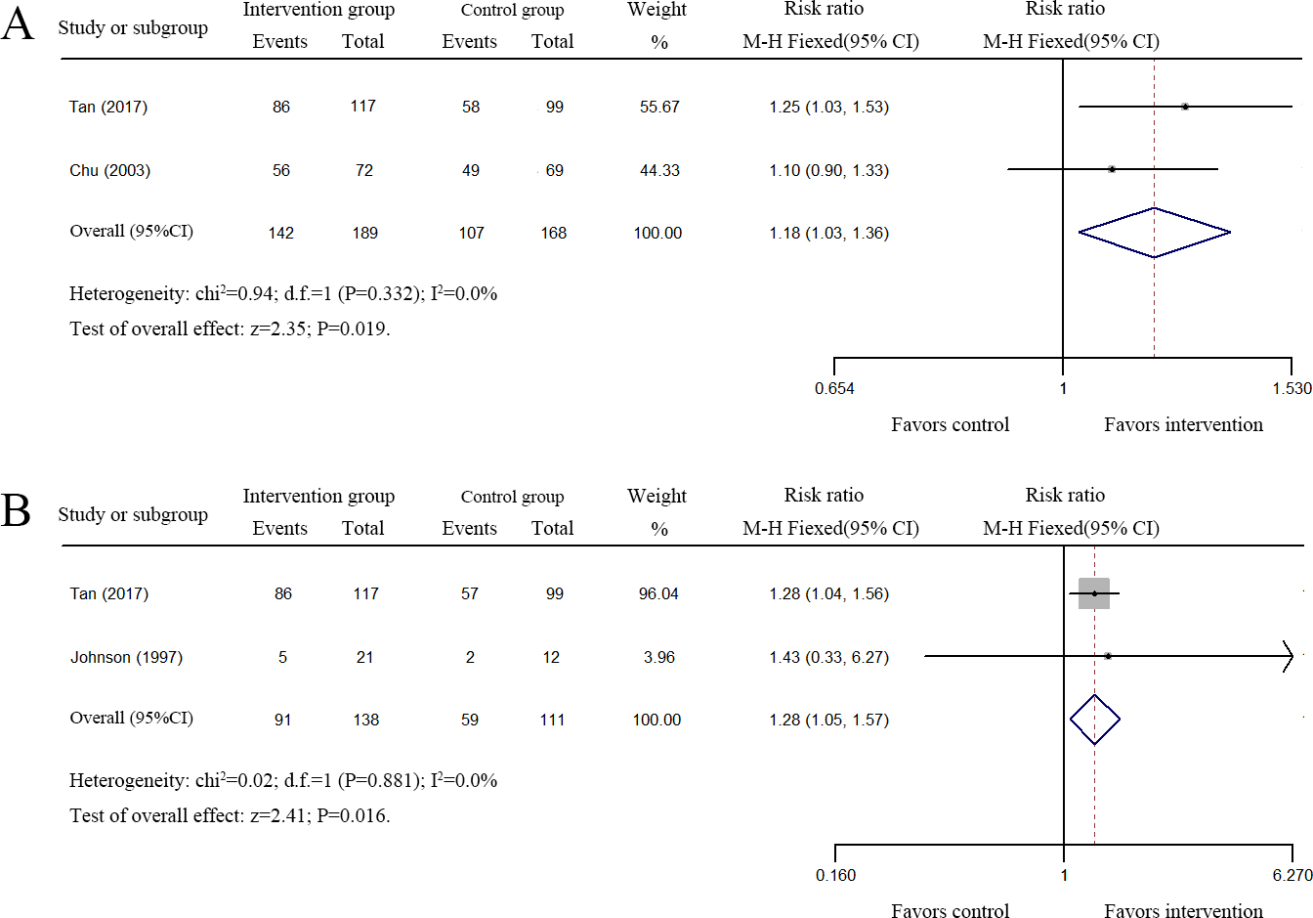
Forest plots of the effect of rhuIL-2 treatment on sputum culture conversion. A. Meta-analysis of the sputum culture conversion at the third month of anti-TB treatment. B. Meta-analysis of the sputum culture conversion of MDR-TB patients at the end of anti-TB treatment. Weights are calculated from both fixed and random effects models.

Two studies comprising 235 patients reported that rhuIL-2 treatment did not improve the sputum culture conversion after the first 2 months of anti-TB treatment (S1 and S2 Tables) [30,29]. In contrast, two studies comprising 357 patients reported that rhuIL-2 treatment did improve the sputum culture conversion at the third month of anti-TB treatment (RR, 1.18; 95%CI: 1.03–1.36; I^2^ < 0.01; *P* = 0.019) (Fig 2A, S1 and S2 Tables) [28,30]. In 2017, Tan *et al*. found that patients in the rhIL-2 group achieved sputum culture conversion at the end of 6, 12, 18, and 24 months of treatment, significantly higher than that of the patients in the control group (S1 Table) [28]. In 1977, Johnson *et al*. reported that conversion from a positive culture at the start of the study to a negative culture at the end of rhuIL-2 treatment occurred in four daily treated patients, in one pulse-treated patient, and in two placebo-treated patients [18].

A pooled analysis to assess the changes in the sputum culture conversion of the MDR-TB patients at the end of anti-TB therapy was also performed. Two eligible studies comprising 249 MDR-TB patients were assessed [18,28]. As shown in Fig 2B, rhuIL-2 treatment did improve the sputum culture conversion of MDR-TB patients (RR, 1.28; 95%CI: 1.05–1.57; I^2^ < 0.01; *P* = 0.016).

Together, these results suggested that rhuIL-2 treatment could significantly improve the sputum culture conversion of TB patients treated for at least 3 months and improve the sputum culture conversion of MDR-TB patients at the end of anti-TB therapy.

### Sputum smear conversion

Three studies comprising 446 patients reported the sputum smear, which was assessed by direct microscopy at different stages (from 1 week to 24 months) of anti-TB treatment (S3 Table) [30,18,28].

In 1977, Johnson *et al*. reported that there was a decreasing trend of a positive rate of sputum smear in the MDR-TB patients receiving continuous daily rhuIL-2 therapy [18]. In 2003, Chu *et al*. suggested that the sputum smear conversion was increased at the end of 1–2 months of treatment with rhuIL-2 [30]. In 2017, Tan *et al*. found that patients in the rhIL-2 group achieved sputum smear conversion at the end of 6, 12, 18, and 24 months of treatment, notably higher than that of patients in the control group [28]. At 3 months of anti-TB therapy, the rate of sputum smear conversion was slightly increased by rhuIL-2 treatment in the studies conducted by Tan *et al*. (69.23% vs. 59.60%) and Chu *et al*. (82.52% vs. 78.00%) [28,30].

**Fig 3:**
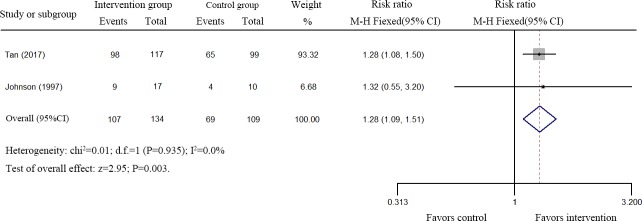
Forest plots of the effect of rhuIL-2 treatment on sputum smear conversion of MDR-TB patients.

Two eligible studies comprising 249 MDR-TB patients were assessed [18,28]. As shown in Fig 3, rhuIL-2 treatment improved the sputum culture conversion of MDR-TB patients (RR, 1.28; 95%CI: 1.09–1.51; I^2^ < 0.01; P = 0.003) at the end of anti-TB treatment.

Together, these results suggest that rhuIL-2 treatment could increase the sputum smear conversion of TB patients as well as improve the sputum smear conversion of MDR-TB patients at the end of anti-TB therapy.

### Radiographic changes

Four studies comprised 547 patients reported the rate of radiographic changes, which was assessed by chest imaging as their diagnostic criteria, during anti-TB treatment [20,29,18,30]. As shown in S4 Table, both the number of repeated times and radiographic response definitions were different. Due to this reason, pooled data analysis was not performed.

Three of the four studies revealed that treatment with rhuIL-2 as adjunctive immunotherapy did not improve the radiographic changes of TB patients (S5 Table) [29,18,30]. In 2017, Tan *et al*. found that rhIL-2 treatment did improve the lung focus resolution of TB patients, but it did not affect the lung cavity closure (S5 Table) [28].

Based on the above results, we conclude that radiographic changes may not be improved by rhuIL-2 as an adjunctive immunotherapeutic agent.

### Leukocyte phenotype changes

Leukocyte phenotypes were stained with the corresponding monoclonal antibodies and analyzed by flow cytometry at different time points in all of the included studies. However, pooled data analysis was not performed.

In 1977, Johnson *et al*. reported that daily rhuIL-2 treatment could increase the mean number of circulating natural killer (NK) cells (CD16^+^/CD56^+^) at the post-study timepoint, compared to the baseline NK cell numbers in the MDR-TB patients [18]. In 2003, Johnson *et al*. reported that the median percentage of CD4^+^/CD25^+^ T lymphocytes was greater after 2 and 6 weeks of anti-TB treatment in subjects receiving rhulL-2 than in those receiving placebo [29]. In the same year, Chu *et al*. suggested that compared with baseline measurements, rhuIL-2 treatment significantly increased the mean numbers of CD4^+^ and NK cells as well as the percentages of CD4^+^/CD8^+^ cells at 3 and 7 months [30]. In 2017, Tan *et al*. found that rhIL-2 treatment increased the percentages of CD3^+^CD8^-^interferon (IFN)-γ^+^ and CD4^+^CD25^+^Foxp3^+^ cells as well as decreased the percentage of CD3^+^CD8^-^IL-17^+^ cells compared to those in the control group at 6 months and 12 months (S6 Table) [28].

From the above results, we concluded that rhuIL-2 treatment could promote the proliferation and conversion of leukocytes.

### Publication bias

**Fig 4:**
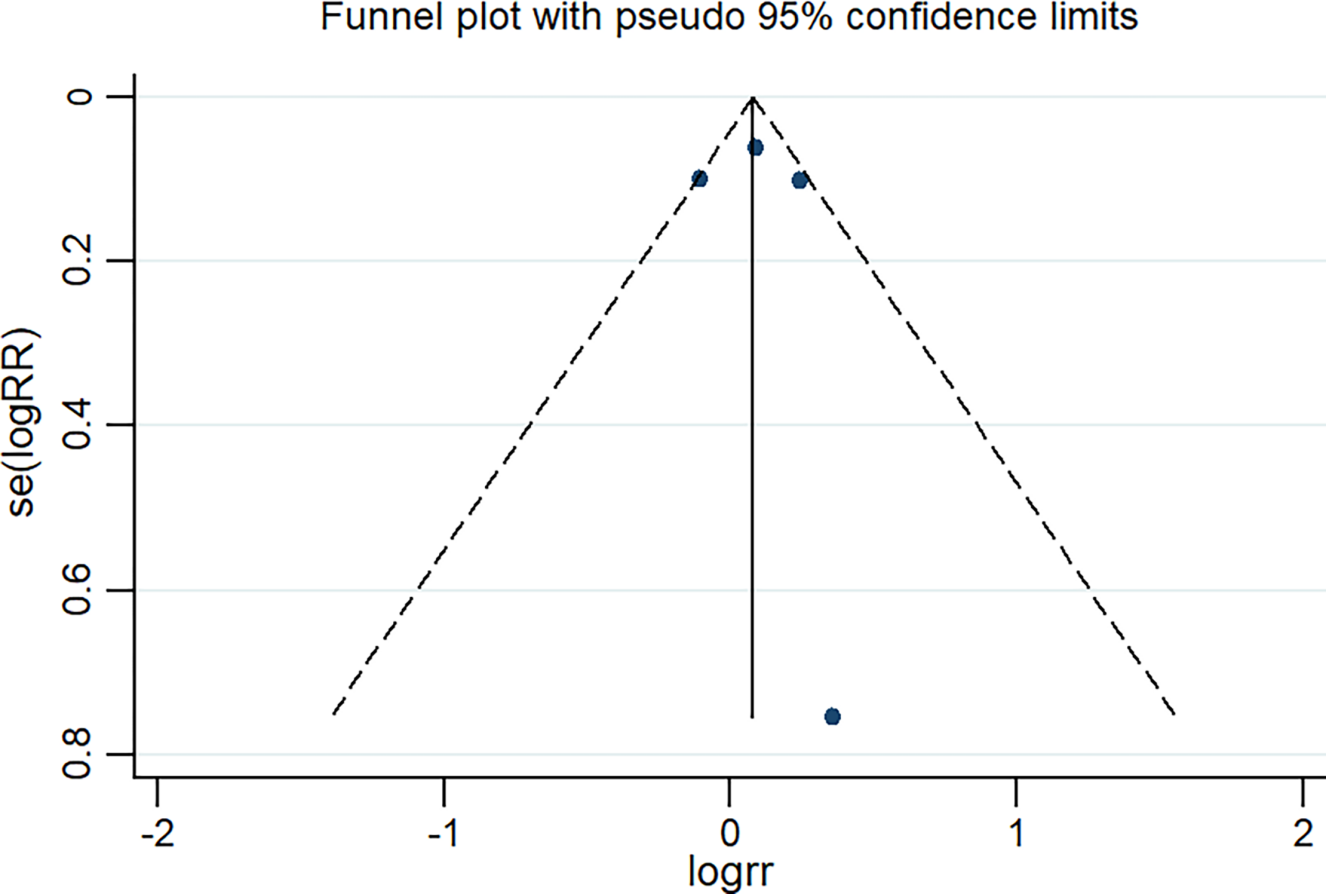
Funnel plot of the four eligible studies that reported sputum culture conversion in pulmonary tuberculosis patients.

Publication bias was evaluated by a funnel plot. As shown in Fig 4, a funnel plot of the sputum culture conversion was established, and there was no publication bias (z = 0.34 [continuity corrected], Pr > |z| = 0.734 > 0.05).

## Discussion

In the past 30 years, IL-2-based adjunctive immunotherapy has attracted increasing attention. To the best of our knowledge, this is the first systemic review and meta-analysis to explore the effect of adjunctive therapy with rhuIL-2 during the treatment of patients with TB. Four clinical RCT studies comprising 656 pulmonary TB patients were included in this study. No publication bias existed. Pooled data analysis showed that treatment of TB patients with rhuIL-2 expanded the proliferation and conversion of CD4^+^ and NK cells as well as improved the sputum culture (at 3 months and later) and smear conversion of TB. Unfortunately, rhuIL-2 treatment did not enhance the radiographic changes.

Recent studies have proven that protective immunity against *M*. *TB* is based on cell-mediated immunity involving CD4 and CD8 T cells [31–33]. It is widely accepted that CD4 T cells play an important role in protective immunity against TB by secreting IFN-γ, tumor necrosis factor-α, and IL-2 [34–36]. However, the contribution of CD8 T cells to immunity against TB is still under debate. Some research suggests that CD8 T cells have a significant role in the control of *M*. *TB* infection [37–39], whereas others disagree [40–42]. Many studies have suggested that the use of IL-2 in vitro can restore some of the anti-bacterial reactivity of T cells [43]. In this study, we demonstrated that rhuIL-2-based adjunctive immunotherapy could expand the proliferation and conversion of CD4^+^ cells, but it did not affect the changes of CD8^+^ T cells.

NK cells are specialized lymphocytes of the innate immune system that are activated during the early response to pulmonary TB through NK cell-derived IFN-γ, which differentially regulates T cell-independent resistance and granulocyte function in *M*. *TB* infection [44–46]. Many studies have shown that IL-2 can augment the cytotoxic activity of NK cells [47]. We report here that the NK cell count was increased by rhuIL-2 treatment.

Based on the above results, we conclude that rhuIL-2-based adjunctive immunotherapy can expand the proliferation and conversion of CD4^+^ and NK cells, improve the function of host immunity, and manipulate the evolution and progression of pulmonary TB.

A negative acid fast bacilli test result in both the sputum smear and culture is a widely accepted technique to determine the effectiveness of treatment and the infectivity of a pulmonary TB patient [48–50]. Sputum smear- and culture-negative conversion rates are considered as prognostic markers of anti-TB treatment [51–53]. In this study, both sputum smear and culture conversion were assessed at different months of treatment. Three of the included trials reported that the sputum smear-negative conversion rates were significantly improved by rhuIL-2 treatment [30,18,28]. Meanwhile, rhuIL-2 treatment for at least 3 months significantly improved the sputum culture conversion of TB patients [28,29,18,30]. However, it has been reported that rhuIL-2 treatment did not enhance bacillary clearance in HIV-seronegative adults with drug-susceptible TB [29].

We also found that rhuIL-2 treatment could improve the sputum smear and culture conversion rates of MDR-TB patients in two of the included studies [18,28]. Therefore, the sputum smear and culture conversion rates could be improved by treating TB patients with rhuIL-2.

Pulmonary TB always produces a broad spectrum of radiographic abnormalities. Chest X-ray is the primary radiologic evaluation method for suspected or confirmed pulmonary TB [54]. In this meta-analysis, all of the included studies applied chest X-ray to evaluate the resolution of TB lesions during anti-TB therapy [29,18,30,28]. We found that rhlL-2 treatment did not increase the rate of chest radiographic improvement in pulmonary TB patients. However, the most recent study found that patients receiving rhIL-2 tended to have a greater improvement of focus resolution in the short term, compared to those in the control group at the end of anti-TB therapy [28]. More research is required to confirm these findings.

## Study limitations

Several important limitations regarding this study should be noted. First, the patients came from China, Uganda, and South Africa. Therefore, the representation and reliability of the results are poor. Second, we did not evaluate some prospective observational studies that involved rhuIL-2-based adjunctive immunotherapy on TB patients. Third, the protocols of rhuIL-2 intervention (rhuIL-2 source, beginning times, delivery methods, dosages, schedules, and therapy period) of rhuIL-2 were different in each study. Fourth, many methods were used among the studies. For example, one study did not clearly state the randomization methods, and only one study was performed in a double-blind manner, while the remaining three were not. Finally, the diagnostic criteria of the radiographic changes were not unified.

Given these findings, more prospective RCTs with a large sample size and a strict design are necessary in future studies.

## Conclusions

Treating TB with rhuIL-2 could expand the proliferation and conversion of CD4^+^ and NK cells as well as improve the sputum culture (at 3 months and later) and smear conversion of TB. However, rhuIL-2 treatment did not enhance the radiographic changes. Large scale, well-designed, multicenter clinical trials are necessary in the future.

## Supporting information

S1 Text Cochrane search strategy. (PDF)

S1 Table Sputum culture conversion assessment. (DOC)

S2 Table Pooled analysis of sputum culture conversion at different months. (DOC)

S3 Table Sputum smear assessments. (DOC)

S4 Table Radiographic assessments. (DOC)

S5 Table Radiographic changes analysis. (DOC)

S6 Table Immunologic cells changes. (DOC)

S7 Table PRISMA 2009 checklist. (PDF)
