# Supplementary material for: Therapeutic effects of recombinant human interleukin 2 as adjunctive immunotherapy against tuberculosis: A systematic review and meta-analysis
Source: PLoS One. 2018 Jul 19;13(7):e0201025. doi: 10.1371/journal.pone.0201025 (PMC6053227; doi:10.1371/journal.pone.0201025)
Supplement: S1 Text — (PDF) [file pone.0201025.s001.pdf]

#1 MeSH descriptor: [Tuberculosis] explode all trees
#2 active tuberculosis:ti,ab,kw (Word variations have been searched)
#3 chronic tuberculosis:ti,ab,kw (Word variations have been searched)
#4 minimal tuberculosis:ti,ab,kw (Word variations have been searched)
#5 minimum tuberculosis:ti,ab,kw (Word variations have been searched)
#6 mycobacterium tuberculosis infection:ti,ab,kw (Word variations have been searched)
#7 endocrine:ti,ab,kw (Word variations have been searched)
#8 tuberculous infection:ti,ab,kw (Word variations have been searched)
#9 tuberculous lesion:ti,ab,kw (Word variations have been searched)
#10 #1 OR #2 OR #3 OR #4 OR #5 OR #6 OR #7 OR #8 OR #9
#11 MeSH descriptor: [interleukin 2] explode all trees
#12 bioleukin:ti,ab,kw (Word variations have been searched)
#13 il 2:ti,ab,kw (Word variations have been searched)
#14 interleukin ii:ti,ab,kw (Word variations have been searched)
#15 interleukin-2:ti,ab,kw (Word variations have been searched)
#16 lymphocult t hp:ti,ab,kw (Word variations have been searched)
#17 lymphocyte mitogenic factor:ti,ab,kw (Word variations have been searched)
#18 t cell growth factor:ti,ab,kw (Word variations have been searched)
#19 t cell growth factor 2:ti,ab,kw (Word variations have been searched)
#20 t lymphocyte growth growth factor 2:ti,ab,kw (Word variations have been searched)
#21 t lymphocyte growth growth factor:ti,ab,kw (Word variations have been searched)
#22 rhulL-2:ti,ab,kw (Word variations have been searched)
#23 recombinant human IL-2:ti,ab,kw (Word variations have been searched)
#24 #11 OR #12 OR #13 OR #14 OR #15 OR #16 OR #17 OR #18 OR #19 OR #20 OR #21 OR #22 OR #23
#25 MeSH descriptor: [randomized controlled trial] explode all trees

#26 controlled clinical trial:ti,ab,kw (Word variations have been searched)
#27 randomized:ti,ab,kw (Word variations have been searched)
#28 placebo:ti,ab,kw (Word variations have been searched)
#29 drug therapy:ti,ab,kw (Word variations have been searched)
#30 randomly:ti,ab,kw (Word variations have been searched)
#31 trial:ti,ab,kw (Word variations have been searched)
#32 groups:ti,ab,kw (Word variations have been searched)
#33 #23 OR #24 OR #25 OR #26 OR #27 OR #28 OR #29 OR #30 OR #31 OR #32
#34 #10 AND #24 AND #33
