# Supplementary material for: Therapeutic effects of recombinant human interleukin 2 as adjunctive immunotherapy against tuberculosis: A systematic review and meta-analysis
Source: PLoS One. 2018 Jul 19;13(7):e0201025. doi: 10.1371/journal.pone.0201025 (PMC6053227; doi:10.1371/journal.pone.0201025)
Supplement: S1 Table — (DOC) [file pone.0201025.s002.doc]

**S1 Table. Sputum culture conversion assessment**

| **Follow-up**  **times** | **Tan et al. [28]** | | | **Johnson et al. [29]** | | | **Chu et al. [30]** | | |
| --- | --- | --- | --- | --- | --- | --- | --- | --- | --- |
|  | **rhuIL-2** | **control** | **P value** | **rhuIL-2** | **control** | **P value** | **rhuIL-2** | **control** | **P value** |
| 1 months |  |  |  | 8/47 | 14/47 | 0.14 | 24/72 | 5/69 | ＜0.001 |
| 2 months |  |  |  | 36/47 | 40/47 | 0.29 | 50/72 | 31/69 | 0.003 |
| 3 months | 86/117 | 58/99 | 0.080 |  |  |  | 56/72 | 49/69 | 0.358 |
| 6 months | 86/117 | 58/99 | 0.020 |  |  |  |  |  |  |
| 7 months |  |  |  |  |  |  | 66/72 | 58/69 | 0.161 |
| 12 months | 86/117 | 56/99 | 0.009 |  |  |  |  |  |  |
| 18 months | 88/117 | 55/99 | 0.002 |  |  |  |  |  |  |
| 24 months | 89/117 | 57/99 | 0.004 |  |  |  |  |  |  |
