# Supplementary material for: Therapeutic effects of recombinant human interleukin 2 as adjunctive immunotherapy against tuberculosis: A systematic review and meta-analysis
Source: PLoS One. 2018 Jul 19;13(7):e0201025. doi: 10.1371/journal.pone.0201025 (PMC6053227; doi:10.1371/journal.pone.0201025)
Supplement: S2 Table — (DOC) [file pone.0201025.s003.doc]

**S2 Table. Pooled analysis of sputum culture conversion at different months.**

|  | Studies | χ^2^ | df | P value | Model | I^2^ | RR | 95% CI | Z value | P value |
| --- | --- | --- | --- | --- | --- | --- | --- | --- | --- | --- |
| 1 months | Johnson et al. [29]  Chu et al. [30] | 12.23 | 1 | 0.000 | Random | 91.8 | 1.60 | 0.20~12.76 | 0.44 | 0.658 |
| 2 months | Johnson et al. [29]  Chu et al. [30] | 10.37 | 1 | 0.001 | Random | 90.4 | 1.17 | 0.65~2.09 | 0.52 | 0.603 |
| 3 months | Tan et al. [28]  Chu et al. [30] | 0.94 | 1 | 0.332 | Fixed | 0.0 | 1.18 | 1.03~1.36 | 2.35 | 0.019 |
