# Supplementary material for: Therapeutic effects of recombinant human interleukin 2 as adjunctive immunotherapy against tuberculosis: A systematic review and meta-analysis
Source: PLoS One. 2018 Jul 19;13(7):e0201025. doi: 10.1371/journal.pone.0201025 (PMC6053227; doi:10.1371/journal.pone.0201025)
Supplement: S3 Table — (DOC) [file pone.0201025.s004.doc]

**S3 Table.** **Sputum smear assessments.**

| **Follow-up**  **times** | **Tan et al. [28] ^*^** | | | **Chu et al. [30] ^*^** | | | **Johnson et al. [18] ^#^** | | | |
| --- | --- | --- | --- | --- | --- | --- | --- | --- | --- | --- |
|  | **rhuIL-2** | **control** | **P value** | **rhuIL-2** | **control** | **P value** | **DRG** | **PRG** | **control** | **P value** |
| 1 weeks |  |  |  |  |  |  | 10/12 | 8/9 | 9/12 | 0.706 |
| 2 weeks |  |  |  |  |  |  | 6/12 | 8/9 | 8/12 | 0.174 |
| 3 weeks |  |  |  |  |  |  | 6/12 | 8/9 | 7/12 | 0.166 |
| 4 weeks |  |  |  |  |  |  | 5/12 | 8/9 | 7/12 | 0.089 |
| 1 months |  |  |  | 52/103 | 21/100 | ＜0.001 |  |  |  |  |
| 5 weeks |  |  |  |  |  |  | 3/8 | 6/9 | 7/10 | 0.324 |
| 6 weeks |  |  |  |  |  |  | 2/6 | 7/9 | 5/10 | 0.209 |
| 7 weeks |  |  |  |  |  |  | 2/5 | 6/8 | 6/8 | 0.350 |
| 8 weeks |  |  |  |  |  |  | 0/5 | 5/6 | 5/7 | 0.012 |
| 2 months |  |  |  | 78/103 | 52/100 | ＜0.001 |  |  |  |  |
| 9 weeks |  |  |  |  |  |  |  |  |  |  |
| 3 months | 81/117 | 59/99 | 0.140 | 85/103 | 78/100 | 0.418 |  |  |  |  |
| 6 months | 89/117 | 59/99 | 0.009 |  |  |  |  |  |  |  |
| 7 months |  |  |  | 96/103 | 88/100 | 0.203 |  |  |  |  |
| 12 months | 93/117 | 63/99 | 0.009 |  |  |  |  |  |  |  |
| 18 months | 94/117 | 64/99 | 0.009 |  |  |  |  |  |  |  |
| 24 months | 98/117 | 65/99 | 0.002 |  |  |  |  |  |  |  |

DRG: Daily rhuIL-2 groups; PRG: Pulse-therapy rhuIL-2 group**.**

**^*^** Sputum smear conversion, **^#^** The positive rate of sputum smear.
