# Supplementary material for: Therapeutic effects of recombinant human interleukin 2 as adjunctive immunotherapy against tuberculosis: A systematic review and meta-analysis
Source: PLoS One. 2018 Jul 19;13(7):e0201025. doi: 10.1371/journal.pone.0201025 (PMC6053227; doi:10.1371/journal.pone.0201025)
Supplement: S4 Table — (DOC) [file pone.0201025.s005.doc]

**S4 Table. Radiographic assessments.**

| **Source** | **Years** | **Methods** | **Repeated times** | **Objective items** | **Objective criterion** |
| --- | --- | --- | --- | --- | --- |
| Tan et al. [28] | 2017 | Chest radiographs | 3, 6, 12, 18, 24 months | Lung focus resolution  Lung cavity closure | Four grades: marked resolution, resolution, no changes, deterioration. |
| Johnson et al. [29] | 2003 | Chest X-rays | 1, 2, 6, and 12 months | One or more severity grades | Standardized scheme which published by Falk et al in 1969. |
| Chu et al. [30] | 2003 | Chest X-rays | Once every month | Lung focus resolution  Lung cavity closure | Standardized scheme revised by CSTB in 1982. |
| Johnson et al. [18] | 1997 | Chest radiograms | Baseline: 1 week prior to study.  Reexamination: 1 week following the last rhuIL-2 injection. | A semi-quantitative score based on the presence of infiltrates and the size of cavities in each of the six zones of the lung. | Read by an onsite physician and by two independent and treatment blinded physicians. |

CSTB: Chinese Society for Tuberculosis, Chinese Medical Association
