# Supplementary material for: Therapeutic effects of recombinant human interleukin 2 as adjunctive immunotherapy against tuberculosis: A systematic review and meta-analysis
Source: PLoS One. 2018 Jul 19;13(7):e0201025. doi: 10.1371/journal.pone.0201025 (PMC6053227; doi:10.1371/journal.pone.0201025)
Supplement: S5 Table — (DOC) [file pone.0201025.s006.doc]

**S5 Table.** **Radiographic changes analysis**.

| **Follow-up**  **times** | **Tan et al. [28]** | | | **Johnson et al. [29]** | | | **Chu et al. [30]** | | |
| --- | --- | --- | --- | --- | --- | --- | --- | --- | --- |
|  | **rhuIL-2** | **control** | **P value** | **rhuIL-2** | **control** | **P value** | **rhuIL-2** | **control** | **P value** |
| End of TB treatment |  |  |  |  |  |  |  |  |  |
| Baseline to month 1 |  |  |  | 12/48 | 9/47 | 0.42 |  |  |  |
| Baseline to month 2 |  |  |  | 19/48 | 15/47 | 0.30 |  |  |  |
| Baseline to month 3 | LFR:75/117  LCC:11/95 | LFR:46/99  LCC:3/78 | LFR:0.009  LCC:0.060 |  |  |  | 37/103 | 93/100 | ＜0.01 |
| Baseline to month 6 | LFR:73/117  LCC:15/95 | LFR:39/99  LCC:8/78 | LFR:＜0.001  LCC:0.280 | 34/48 | 35/47 | 0.69 |  |  |  |
| Baseline to month 7 |  |  |  |  |  |  | 66/103 | 96/100 | ＜0.01 |
| Baseline to month 12 | LFR:71/117  LCC:28/95 | LFR:41/99  LCC:13/78 | LFR:0.005  LCC:0.070 | 43/48 | 41/47 | 1.000 |  |  |  |
| Baseline to month 18 | LFR:70/117  LCC:36/95 | LFR:42/99  LCC:17/78 | LFR:0.011  LCC:0.030 |  |  |  |  |  |  |
| Baseline to month 24 | LFR:74/117  LCC:41/95 | LFR:46/99  LCC:22/78 | LFR:0.001  LCC:0.060 |  |  |  |  |  |  |

DRG: Daily rhuIL-2 groups; PRG: Pulse-therapy rhuIL-2 group**;** LFR: Lung focus resolution; LCC: Lung cavities closure.
