# Supplementary material for: Therapeutic effects of recombinant human interleukin 2 as adjunctive immunotherapy against tuberculosis: A systematic review and meta-analysis
Source: PLoS One. 2018 Jul 19;13(7):e0201025. doi: 10.1371/journal.pone.0201025 (PMC6053227; doi:10.1371/journal.pone.0201025)
Supplement: S6 Table — (DOC) [file pone.0201025.s007.doc]

**S6 Table. Immunologic cells changes.**

| **Follow-up times** | Tan et al. [28] | | | Chu et al. [30] | | |
| --- | --- | --- | --- | --- | --- | --- |
|  | rhuIL-2 | control | P value | rhuIL-2 | control | P value |
| **Baseline** |  |  |  |  |  |  |
| **-** **CD3^+^CD8^-^IL-17^+^cells** | 7.45±1.03 | 7.02±1.63 | 0.52 |  |  |  |
| **-****CD4+CD25^+^Foxp3^+^cells** | 3.28±0.55 | 3.32±0.99 | 0.88 |  |  |  |
| **- CD3^+^CD8^-^IFN-γ^+^ cells** | 11.99±3.46 | 12.03±2.93 | 0.92 |  |  |  |
| **-CD_4_** |  |  |  | 35±8 | 35±7 | 0.5509 |
| **-CD_4_/CD_8_** |  |  |  | 1.4±0.4 | 1.3±0.3 | 0.1513 |
| **-NK cell** |  |  |  | 13.6±3.2 | 12.8±1.4 | 0.1616 |
| **3 months** |  |  |  |  |  |  |
| **-CD_4_** |  |  |  | 40±7 | 36±8 | 0.0058 |
| **-CD_4_/CD_8_** |  |  |  | 1.6±0.3 | 1.4±0.2 | 0.0001 |
| **-NK cell** |  |  |  | 15.0±2.1 | 13.2±1.0 | 0.0000 |
| **6 months** |  |  |  |  |  |  |
| **- CD3^+^CD8^-^IL-17^+^cells** | 6.25±1.15 | 4.83±1.46 | 0.02 |  |  |  |
| **-CD4+CD25^+^Foxp3^+^cells** | 3.19±0.72 | 2.92±0.73 | 0.21 |  |  |  |
| **-** **CD3^+^CD8^-^IFN-γ^+^ cells** | 12.73±3.05 | 14.36±2.50 | 0.04 |  |  |  |
| **7 months** |  |  |  |  |  |  |
| **-CD_4_** |  |  |  | 41±8 | 38±8 | 0.0022 |
| **-CD_4_/CD_8_** |  |  |  | 1.7±0.3 | 1.4±0.3 | 0.0000 |
| **-NK cell** |  |  |  | 15.0±1.5 | 13.7±0.8 | 0.0000 |
| **12 months** |  |  |  |  |  |  |
| **- CD3^+^CD8^-^IL-17^+^cells** | 5.07±1.94 | 3.38±1.56 | <0.01 |  |  |  |
| **-CD4+CD25^+^Foxp3^+^cells** | 2.98±0.73 | 2.39±0.66 | <0.01 |  |  |  |
| **- CD3^+^CD8^-^IFN-γ^+^ cells** | 15.07±3.32 | 17.23±1.99 | 0.01 |  |  |  |
